# Is the superior verbal memory span of Mandarin speakers due to faster rehearsal?

**DOI:** 10.3758/s13421-017-0770-8

**Published:** 2017-11-06

**Authors:** Sven L. Mattys, Alan Baddeley, Danijela Trenkic

**Affiliations:** 1 Department of Psychology, University of York, York, UK; 2 Department of Education, University of York, York, UK

**Keywords:** Chinese memory span, Verbal rehearsal, Digit span, Articulation speed, Cross-linguistic working memory

## Abstract

It is well established that digit span in native Chinese speakers is atypically high. This is commonly attributed to a capacity for more rapid subvocal rehearsal for that group. We explored this hypothesis by testing a group of English-speaking native Mandarin speakers on digit span and word span in both Mandarin and English, together with a measure of speed of articulation for each. When compared to the performance of native English speakers, the Mandarin group proved to be superior on both digit and word spans while predictably having lower spans in English. This suggests that the Mandarin advantage is not limited to digits. Speed of rehearsal correlated with span performance across materials. However, this correlation was more pronounced for English speakers than for any of the Chinese measures. Further analysis suggested that speed of rehearsal did not provide an adequate account of differences between Mandarin and English spans or for the advantage of digits over words. Possible alternative explanations are discussed.

Digit span, first developed by Jacobs (1887), continues to play an important role in both clinical and educational psychometric tests. For example, low digit span has been found in children with delayed reading (Gathercole, Briscoe, Thorn, Tiffany, & ALSPAC Study Team, 2008) and to be associated with slow acquisition of vocabulary (Gathercole & Baddeley, 1989, 1990). Low digit span is also predictive of difficulty in second language learning (Atkins & Baddeley, 1998).

At a theoretical level, despite their apparent simplicity, digit and related verbal serial recall measures have proved both complex and fruitful. They potentially throw light on two important issues: The way in which serial order is stored and the potential for increasing capacity by binding items into chunks with span determined by number of chunks rather than items (Miller, 1956). Chunking has in turn been shown to be potentially influenced by long-term memory, as in the case of sequences of letters or words where the closer the sequence approximates the structure of the native language, the better the performance (Baddeley, 1971; Miller & Selfridge, 1950; Thorn & Gathercole, 1999; Tulving & Patkau, 1962). More recently, an analogous effect of prior knowledge on immediate memory for digits has been shown by Jones and Macken (2015).

Theoretically oriented studies have tended to fall into two separate but related approaches. Some studies are interested in linking immediate memory to long-term memory. These tend to focus on the influence of existing language knowledge on memory performance. Other studies attempt to analyse the underlying mechanisms for short-term encoding and maintenance, typically minimising general language effects by using sequences of unrelated digits or letters for which the influence of long-term memory is reduced. One such approach is based on the Baddeley and Hitch (1974) multicomponent working memory model. This assumes a subcomponent of working memory, the phonological loop, comprising a temporary store in which phonological/acoustic traces fade over a matter of seconds, together with an articulatory rehearsal system capable of maintaining a limited amount of spoken material. Evidence for the rehearsal system comes from the word-length effect, whereby immediate serial recall of words is a function of the number of syllables of the words in the list. In a study by Baddeley, Thomson, and Buchanan (1975), sequences of five monosyllables showed more than 90% correct recall compared to around 50% for five-syllable items matched for semantic category. They explained the word-length effect by assuming that long words take more time to articulate, and that this in turn leads to greater decay of the underlying memory trace. Further evidence for a subvocal rehearsal interpretation of this effect comes from the fact that the word-length effect is lost under articulatory suppression, the requirement to repeatedly utter an unrelated word, such as *the* during presentation and test (Baddeley et al., 1975; D. J. Murray, 1968).

While the syllabic word-length effect itself is robust (Tehan, Hendry, & Kocinski, 2001), its interpretation remains controversial. Baddeley et al. (1975) attributed the effect to trace decay, with longer words taking longer to rehearse, hence allowing more decay. This contrasts with an interference hypothesis, which would argue that more syllables lead to greater interference and poorer recall. Evidence for temporal decay came from a study in which disyllabic words with short pronunciation times (e.g., *bishop, wicket*) were better retained than disyllabic words with longer pronunciation times (e.g., *Friday, harpoon*). However, other studies using a different sample of words failed to replicate this effect (e.g., Lovatt, Avons, & Masterson, 2000; Service, 1998). Interpretation is further complicated by between-word coarticulation effects. Coarticulation effects have been shown for long-term learning, whereby learning a nonsense pair like *ZIL–TOV*, which is easy to articulate, was easier than learning *TOV–ZIL*, which involves an atypical articulatory transition between items (Baddeley, 1964). The relevance of coarticulation was also demonstrated as a potentially important factor in comparing memory spans across languages by A. Murray and Jones (2002), who showed that the immediate memory span for Welsh is shorter than that for English, not because of the time it takes to pronounce individual items but because of greater differences in between-word coarticulation. Such effects are also relevant to the observation that practice with nonwords increases span, with the practice effect being more strongly reflected in an improvement in coarticulation than in time to pronounce the nonwords themselves (Woodward, Macken, & Jones, 2008).

A further constraint in testing an explanation of the word-length effect in terms of trace decay rather than interference comes from the need to match phonological similarity across sets of items, given that higher similarity leads to poorer immediate serial recall (Baddeley, 1966; Conrad & Hull, 1964). In an attempt to settle the word-length controversy, Mueller, Seymour, Kieras, and Meyer (2003) systematically evaluated the influence of both similarity and articulation rate, studying a range of stimuli that had previously been used to answer this question. They concluded that when the relevant variables were adequately controlled, articulation time was crucial, supporting an interpretation in terms of decay rather than interference. Others, however, have taken issue with the particular measures Mueller et al. used and have argued for an alternative interpretation in terms of either the greater complexity of longer words (Service, 2000) or their greater fragility (Brown & Hulme, 1995; Neath & Nairne 1995). Overall, the difficulty in producing sets that are matched not only on number of syllables but also on frequency, and within-set similarity while differing in spoken duration, makes this method unlikely to produce results that are uncontroversially in favour of either decay or interference. The extensive work done by Mueller et al. on measuring rate of articulation does, however, have relevance for the study that follows. We will return to this when describing the measures of articulation we selected.

Ellis and Hennelly (1980) pointed out the potential importance of the word-length effect for the practical question of comparing digit spans across languages, demonstrating that bilingual Welsh speakers had a longer span in English than in Welsh, a language that has syllabically longer digits. Several other studies have also used bilingual participants to compare performance across languages (e.g., Cheung & Kemper, 1993; Hoosain, 1979). However, although bilingual studies have the advantage of allowing a within-participants design, interpretation is complicated by a possible language imbalance, with digits in a first language potentially being more readily retrieved than digits in a second language (Brown & Hulme, 1992; da Costa Pinto, 1991). This can be avoided by comparing separate groups of native speakers across languages, where knowledge of digits can be assumed to be broadly equivalent. Naveh-Benjamin and Ayres (1986) compared memory span for English, Spanish, Hebrew, and Arabic native speakers and found a systematic association between mean syllabic length of digits, rate of sequence articulation, and memory span. Broadly similar results were obtained by Elliott (1992), who studied digit span for Malay, English, and two Chinese dialects.

In the initial studies, English was found to show the longest span, associated with the fact that all digits, except seven, are monosyllables. However, Hoosain and Salili (1987) observed that pronunciation speed was higher, sound duration of numbers shorter, and span longer in Chinese than in English, an effect that was less prominent in backward span in which factors other than simple maintenance of the digits is required (Hoosain, 1979). A more substantial study was carried out by Stigler, Lee, and Stevenson (1986), who tested digit span in Chinese, American, and Japanese kindergarten, first-grade and second-grade children. They found that, across the age range, the Chinese children remembered at least two more digits on average than the American or Japanese children. They also found the difference to be absent in backward span, and that inducing a grouping strategy helped both Chinese and American children to the same extent, ruling out an interpretation in terms of cultural differences in temporal grouping. Finally, the spoken duration of the longest span was equivalent between Chinese and American children, suggesting an interpretation in terms of speed of articulation. A subsequent study by Chen and Stevenson (1988) compared 4-year-old, 5-year-old, and 6-year-old American and Chinese children, replicating the Chinese advantage in forward span and its absence in backward span. They ruled out interpretations in terms of differential practice, counting systems, or strategies, favouring instead an interpretation in terms of subvocal rehearsal.

In this study, we decided to further test the rehearsal-based interpretation of the Chinese span superiority effect by measuring memory span in Mandarin and English in a group of native Mandarin-speaking students attending a university course for teachers of English as a foreign language in the UK.^1^ We measured their span for digits and for high-frequency words in both Mandarin and English, together with their speed of articulation for both types of material. We then compared their performance to that of a sample of native-English speakers from a matched student population.

Our reasons for including word span in addition to digit span were twofold. First, we wanted to investigate the possibility that the Chinese advantage might be due to greater exposure to digits in Chinese education, which is manifest in the generally high performance of Chinese speakers in arithmetic skills (Geary, Bow-Thomas, Fan, & Siegler, 1993). Second, although we used high-frequency words, one would expect access to word representations to be less overlearned than the more frequently accessed digit representations, making the former slower than the latter. Studies of rehearsal in children by Cowan (1992; Cowan et al., 1994, 1998) distinguish between two aspects of verbal rehearsal, one based on the time it takes to retrieve each spoken item, reflected in between-item pause length, and the other based on the time it takes to articulate each item. Jarrold, Hewes, and Baddeley (2000) studied the effects of word length on memory and articulation rate in 5-year-old, 8-year-old, and 10-year-old children. They found that developmental improvement in memory span was associated with shortening interword gaps, suggesting improved lexical access. In contrast, the word-length effect per se was associated with duration of the spoken items, consistent with time-based forgetting. Unfortunately, however, the data reported below showed that, unlike children, adults appear to be able to overlap articulation and retrieval so that interim pauses no longer occur, hence removing this source of potential information.

## Method

### Participants

These were 36 native Mandarin speakers (32 female, four male; average age = 24 years; range: 22–30 years) and 36 native English speakers (29 female, six male, one unspecified; average age = 21 years; range: 19–26 years). All participants were undergraduate or master’s students at the University of York, UK. For the Mandarin group, the average age of acquisition of English as a second language was 10 years (range: 6–15 years). Those participants had resided in the UK for an average of 8 months (range: 4–24 months). Their average International English Language Testing System (IELTS) score was 6.85 (range: 6.0–8.0), equivalent to level B2/C1 of the Common European Framework of Reference for Languages. Mandarin participants also self-assessed their proficiency in English speaking, reading, listening, and writing on a scale from 1 (*low*) to 10 (*high*). Averages (and ranges) were, respectively, 5.92 (3–8), 7.11 (4–8), 6.83 (4–9), and 5.97 (4–8).

### Materials

The stimuli were audio-recordings of the following: (1) The digits 1 to 9 in English and in Mandarin Chinese and (2) Nine monosyllabic English words and their translation in Mandarin Chinese, also monosyllabic. The words were *cow, duck, wolf, pig, bear, cat, hen, dog, horse*. The English digits and words were spoken by a female native-English speaker and the Chinese digits and words were spoken by a female native-Mandarin speaker. The stimuli were recorded in a sound-attenuated booth. For both types of stimuli, the speakers read several lists, each with a different random order. The most intelligible token of each stimulus was selected and saved as an individual sound file. The average durations of the selected English and Chinese digits were 449 ms and 417 ms, respectively, *t*(8) = 1.15, *p* = .28. The average durations of the English and Chinese words were 474 ms and 509 ms, respectively, *t*(8) = −.96, *p* = .37. It is worth noting that these individual word times do not differ significantly, and, indeed, in the case of words, English pronunciations tend, if anything, to be shorter. This suggests that any significant differences in articulation rate may principally reflect coarticulation effects as found by Woodward et al. (2008) in their English–Welsh comparison.

### Design and procedure

Participants were tested individually. The English participants went through the following sequence of tests: English digit span, English digit articulation speed, English word span, and English word articulation speed. The Chinese participants went through the same sequence of tests for both the Chinese and the English materials. They always started with the Chinese materials. The experimenter, who was English–Mandarin bilingual, gave the instructions in English to the English participants and in Chinese to the Chinese participants.

The digit-span and word-span tasks were identical, except for the stimuli used. For both tasks, each trial consisted of a sequence of stimuli (digits or words) presented auditorily, with 650 ms between stimuli. Immediately after the last stimulus of a sequence, the visual prompt ‘Please repeat now’ was displayed on a computer monitor in front of the participant. Participants repeated the stimuli in the same order they heard them, then pressed the spacebar to move on to the next sequence. The number of stimuli in a sequence increased by one every three sequences, starting with three stimuli, and ending with a maximum of 10 stimuli for the English materials and 12 stimuli for the Chinese materials. The difference in maximum number of stimuli was motivated by pilot data indicating that Chinese participants frequently exceed 10 stimuli in Chinese span tasks. A sequence was scored as recalled correctly if all the stimuli in the sequence were repeated in the correct serial position. No feedback was provided. The span task was terminated when the participant failed all three sequences of a given length. A participant’s span was calculated by averaging the last three sequences that he or she had repeated correctly. The maximum number of stimuli was never reached by any of the participants.

As shown by Mueller et al. (2003), it is important to take care in selecting the way in which articulation rate is measured. Our aim was to simulate as closely as possible the covert articulation that would occur in our task. We therefore required the speeded articulation of several items from the relevant set rather than single items varying across trials so as to avoid the danger of using a few atypical sequences. A pilot study suggested that the overt articulation of four consecutive digits or words could lead to hesitations in some participants, making reliable duration measurement difficult. We therefore opted for groups of three items for both words and digits. We used the same material for memory and articulation measures, as in the original Baddeley et al. (1975) study which produced a correlation of .68 between span and reading rate. Other studies have used materials that differ between the articulation and memory conditions, presumably attempting to identify a general measure of articulation speed, typically resulting in a more modest correlation with digit span, with Cowan et al. (1998) finding correlations between digit span and reciting the alphabet or counting from 1 to 10 in the region of .25, while Tehan and Lalor (2000), using word repetition, alphabet recitation, and counting between 20 and 40, found correlations ranging from .09 to .47. A later study by Tehan, Fogarty, and Ryan (2004), again using a range of articulation measures of material other than that to be recalled, found correlations around .25. By using the same material for articulation and memory, we hoped to replicate the higher correlation reported by Baddeley et al. (1975).

The digit and word articulation tasks were identical, except for the stimuli used. For both tasks, each trial started with a 150-ms beep followed by a sequence of three stimuli (digits or words) presented auditorily, with 100 ms between stimuli. The sequence was immediately followed by a visual prompt reading ‘Repeat once’. This single repetition was meant to ensure that the participants had properly heard and encoded the three stimuli. After they had repeated the three stimuli, they pressed the spacebar. They then heard a second 150-ms beep, which prompted them to repeat the same three stimuli several times in a row, as fast as possible, and without interruption, until the experimenter asked them to stop (generally after four or five repetitions). There were 12 such trials, each with a different triplet of stimuli. The articulation tasks started with a practice block containing two sequences. Participants’ productions were audio-recorded. The duration of the first three repetitions of each triplet was measured using a sound editor and averaged for each participant. Each average duration was then divided by 9, which was the total number of stimuli/syllables in the measured utterance, to produce an articulation rate index, expressed as number of syllables uttered per second. This was done separately for digits and words.

## Results

**Fig. 1:**
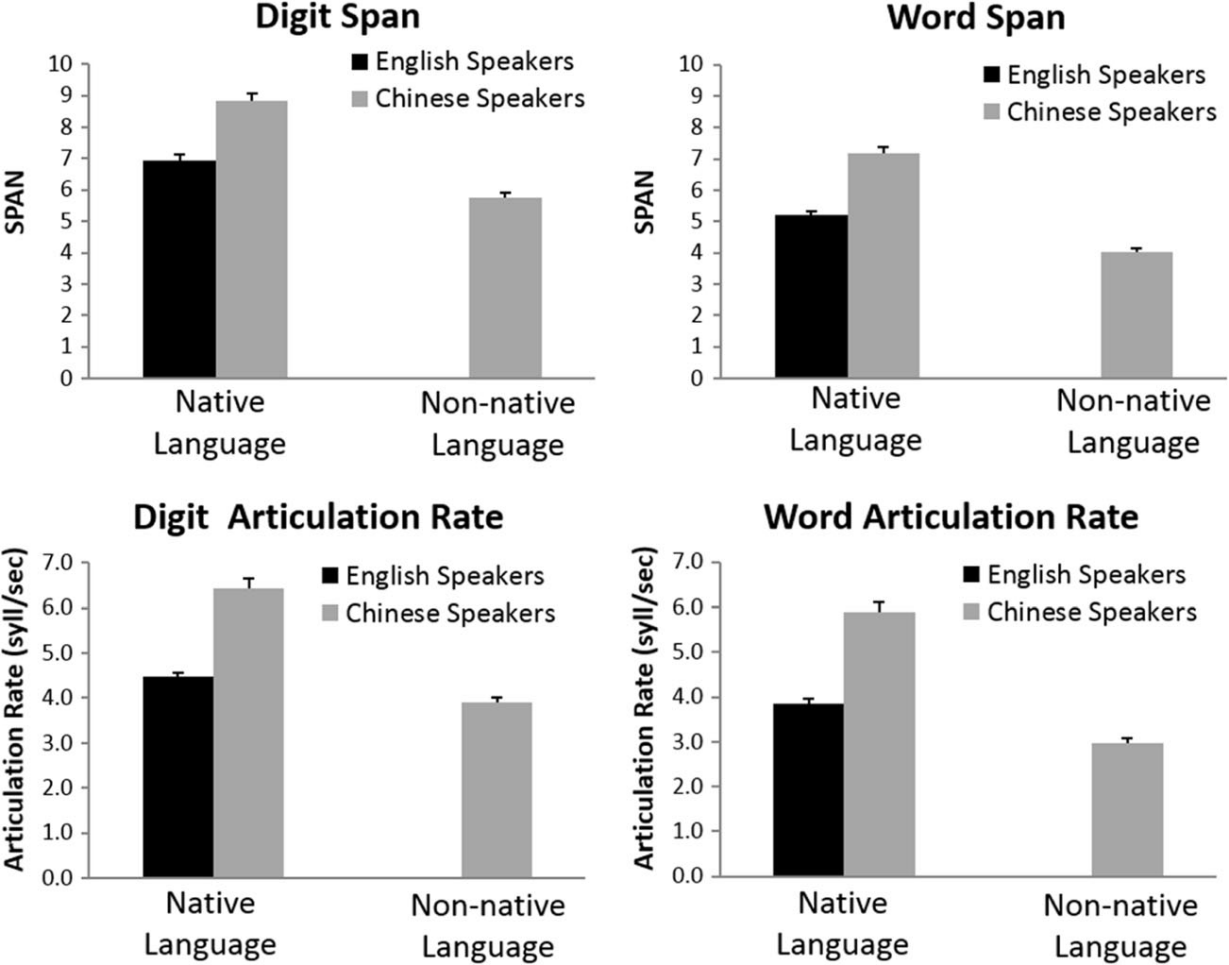
Average digit span and word span for English speakers (on English digits and words) and for Chinese speakers (on Chinese and English digits and words)

The average native and non-native digit and word spans, as well as digit and word articulation rates are plotted in Fig. 1.

### Digit-span and word-span analyses

Chinese participants had a longer digit span in Chinese (*x̅* = 8.84, *SD* = 1.30) than English participants did in English (*x̅* = 6.94, *SD* = 1.03), *t*(70) = 6.87, *p* < .001, *d* = 1.62, which replicates the Chinese advantage reported previously (e.g., Cheung & Kemper, 1993; Hoosain, 1979). Performance of the Chinese participants on English digits (*x̅* = 5.77, *SD* = .93) was significantly below both their performance on Chinese digits, *t*(35) = −17.15, *p* < .001, *d* = 2.85, and the performance of English speakers on English digits, *t*(70) = −5.07, *p* < .001, *d* = 1.19.

The word-span data showed similar patterns. Chinese participants had a longer word span in Chinese (*x̅* = 7.17, *SD* = 1.16) than English participants did in English (*x̅* = 5.20, *SD* = .71), *t*(70) = 8.67, *p* < .001, *d* = 2.04. Performance of the Chinese participants in English (*x̅* = 4.02, *SD* = .69) was significantly below both their performance in Chinese, *t*(35) = −18.14, *p* < .001, *d* = 3.02, and the performance of English speakers in English, *t*(70) = −7.14, *p* < .001, *d* = 1.67.

When the above analyses were run comparing digits and words, digit spans were systematically longer than word spans, all analyses *p*s < .001, but this effect did not interact with the above patterns, all interaction *p*s > .70.

### Digit and word articulation rate analyses

Because of extensive mispronunciations and hesitations, the articulation rate for two Chinese speakers could not be reliably calculated. Therefore, the analyses are based on 34 of the 36 Chinese participants (and all 36 English participants). The articulation rate patterns closely mirrored the memory span patterns. Chinese participants articulated Chinese digits faster (*x̅* = 6.44 syllables per second, *SD* = 1.23) than English participants articulated English digits (*x̅* = 4.47, *SD* = .63), *t*(68) = 8.50, *p* < .001, *d* = 2.01. Articulation of English digits by Chinese participants (*x̅* = 3.89, *SD* = .67) was significantly slower than their articulation of Chinese digits, *t*(33) = −17.47, *p* < .001, *d* = .85, and slower than the articulation of English digits by English participants, *t*(68) = −3.73, *p* < .001, *d* = .89.

The patterns of articulation rates for words were similar to those for digits. Chinese participants articulated Chinese words faster (*x̅* = 5.88, *SD* = 1.34) than English participants articulated English words (*x̅* = 3.84, *SD* = .69), *t*(68) = 8.04, *p* < .001, *d* = 1.80. Articulation of English words by Chinese participants (*x̅* = 2.97, *SD* = .63) was significantly slower than their articulation of Chinese words, *t*(33) = −16.50, *p* < .001, *d* = 2.83, and slower than the articulation of English words by English participants, *t*(68) = −5.50, *p* < .001, *d* = 1.32.

Articulation rates were generally faster for digits than for words, all analyses *p*s < .001. For the Chinese participants, that difference was larger when they spoke English than Chinese, *F*_interaction_(1, 33) = 11.31, *p* = .002, η_p_^2^ = .25, which probably reflects their greater practice in pronouncing highly frequent digits than our specific set of words in non-native English. Likewise, the digit-vs-word articulation difference was larger for the Chinese speakers than the English speakers when the stimuli were in English, *F*_interaction_(1, 68) = 9.96, *p* = .002, η_p_^2^ = .13.

### Relationship between digit span and articulation rate

**Fig. 2:**
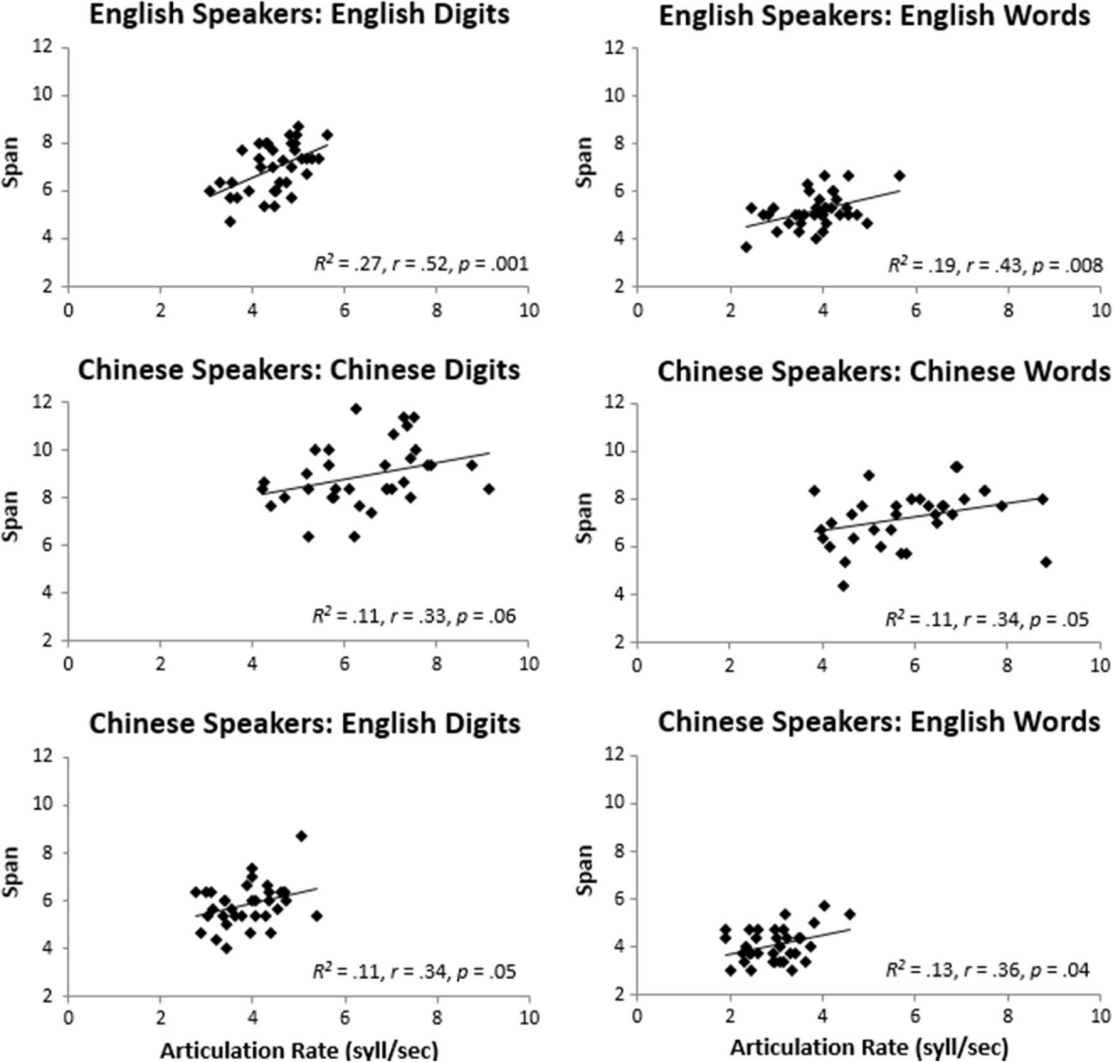
By-participant Pearson correlation coefficients between span and articulation rate for the main conditions

One of the main questions for this study was whether memory span differences are reducible to differences in subvocal rehearsal rates, which we estimated through overt articulation rates. Similarities between span and articulation rate patterns in the previous analyses suggest that the two skills might be interconnected, at least at the group level. Figure 2 shows the by-participant Pearson correlation coefficients between span and articulation rate for each of the main conditions. High memory span was associated with faster articulation rate across the board. However, this relationship was less pronounced in the Chinese group, regardless of the materials and the test language. The contrast between the two groups could reflect either a weaker association between span and articulation in the Chinese group or the additional influence of other factors that potentially contribute to performance.

This raises the question of whether the higher native span for Chinese than for English participants would remain if articulation rate was taken into account. Unfortunately, the significant difference in articulation rates between the two groups and their contrasted span-by-articulation slopes (the central point of our comparison) itself made analyses of covariance unsuitable (e.g., Miller & Chapman, 2001; Schneider, Avivi-Reich, & Mozuraitis, 2015). However, an inspection of the *R* ^2^ values in Fig. 2 showed large proportions of variance in span unexplained by articulation rate (73% to 89%). Therefore, it seems unlikely that articulation rate wholly accounted for the group difference in memory span. A similar conclusion applies to the question of whether the difference between native and non-native spans can be explained by articulation rate differences.

With respect to whether articulation rate can account for the higher span for digits than words, analyses of covariance confirmed that it was unlikely. Unlike the comparison between English and Chinese participants, analyses were licensed by sufficiently similar articulation rates between digits and words (on a group by group basis) and comparable regression slopes between span and articulation rates for digits and words. The digits-vs-words span difference remained for English speakers tested on English stimuli, *F*(1, 34) = 37.86, *p* < .001, η_p_^2^ = .53, for Chinese speakers tested on Chinese stimuli, *F*(1, 32) = 66.66, *p* < .001, η_p_^2^ = .66, and for Chinese speakers tested on English stimuli, *F*(1, 32) = 34.16, *p* < .001, η_p_^2^ = .52. This is consistent with evidence for a range of long-term lexical effects on verbal memory span, notably including word frequency which is clearly higher for digits than for our set of words (Allen & Hulme, 2006; Hulme, Maughan, & Brown, 1991).

## Discussion

Our study had three aims: (1) To revisit the superior native-language digit span shown by Mandarin speakers, (2) to extend it to span for monosyllabic words, and (3) to measure rate of articulation for the two types of material and to test the hypothesis that differences in span were entirely attributable to speed of articulation. We compared native Mandarin-speaking and native English-speaking participants, finding that the observed difference in span generalised to high-frequency words and hence could not be attributed to greater exposure of the Chinese participants to digits during their education.

As anticipated, speed of articulating digits in the native language was consistently faster among the Chinese than the English participants while both groups were faster at articulating digits than words in their native language. Again, as expected, Chinese participants were slower at rehearsing English digits and words than they were at rehearsing in their native language. All of these results suggest a broad association between articulation rate and memory span across the range of materials, in line with earlier studies which have demonstrated a superiority of Chinese over English span (Chen & Stevenson, 1988; Hoosain, 1979; Hoosain & Salili, 1987; Stigler et al., 1986) and in line with studies demonstrating a systematic association between articulation rate and span across a range of languages (Elliott, 1992; Naveh-Benjamin & Ayres, 1986).

A more fine-grain analysis of performance was obtained by assessing the relationship between span and articulation rate across individual participants (Baddeley et al., 1975; Cowan et al., 1994, 1998; Jarrold et al., 2000). Here, we found the expected correlation between span and speed of articulation in English speakers for both digits and words. This effect was less pronounced in Mandarin speakers across all four tested conditions, suggesting that rate of articulation did not provide an adequate explanation of the substantial group differences. A similar conclusion was reached for the difference in span between digits and words in both languages.

In interpreting our results, it is important to make two points. First, speed of articulation appears to play an important role in span performance in our native English speakers and, to a smaller extent, in our Chinese participants, although it does not appear to account for the superior span of the latter group. Second, although verbal rehearsal can play an important role in supporting immediate verbal serial recall, it only provides part of the standard account, serving as a means of refreshing memory traces within the phonological store. Suppressing articulation and using visual presentation removes the influence of subvocal rehearsal, as shown by the resulting abolition of the phonological similarity effect, a classic marker for the involvement of the phonological short-term store (Baddeley, Lewis, & Vallar, 1984). However, although articulatory suppression reduces digit span by around two items, it does not totally disrupt performance. For example, Chincotta and Underwood (1997) found a reduction in digit span under suppression from around 8.9 to 6.2 digits (as estimated from their figure) for Chinese participants and from 7.5 to 5.2 for English participants. The residual span items are assumed to come from sources other than subvocal rehearsal. Hence, our results suggest that the difference between Chinese and English memory spans is based on enhanced storage capacity, in addition to any advantage gained by more rapid rehearsal, raising the question of how this superiority might occur.

The fact that verbal span remains with visual presentation when subvocal rehearsal is prevented indicates a contribution to performance from other nonarticulatory cues, possibly visual, acoustic, or lexical. It seems likely that the extent to which these are used may reflect cultural differences. We understand, for example, that there is a much greater emphasis on verbal rote memory in Chinese culture as indicated for instance in popular TV contests based in rote memory performance. This, in turn, may reflect the much greater demand on verbal rote learning made by the need to learn the names of many hundreds of logographic characters in contrast to the 26 letter names in the alphabet. If this is the case, the greater memory span for Chinese speakers could be based either on an increase in the capacity of the phonological store or possibly on the development of rehearsal processes that extend beyond that of subvocal rehearsal used in English and related languages.

One possible candidate is the process of rehearsal known as ‘refreshing’, whereby paying attention to the memory representation of an item will prolong its storage (Barrouillet & Camos, 2015; Lewandowsky & Oberauer, 2008). The multicomponent model of working memory regards this as the principal mode of rehearsal for systems other than the phonological loop (Baddeley, 2012) for which articulation tends to be the dominant means of maintenance. However, articulatory rehearsal is assumed to be an optional strategy (Campoy & Baddeley, 2008). Furthermore, while articulatory suppression clearly impairs short-term memory, it does not prevent accurate phonological judgements, with participants able to decide whether pairs of words such as *slay* and *sleigh* are homophones rapidly and accurately (Baddeley & Lewis, 1981; Besner, 1987). One possibility, therefore, is that extensive practice in rote learning may have encouraged the parallel use of both articulation and refreshing in retaining items in short-term memory. Following the Baddeley and Lewis (1981) separation between an articulatory code (the ‘inner voice’) and an acoustic code (the ‘inner ear’), English speakers appear to rely principally on articulation for sequences of around span length, abandoning it for longer sequences (Salamé & Baddeley, 1986). It is possible that Chinese speakers have learned to use both articulation and refreshing simultaneously.

If the extensive rote learning required by the need to memorise many characters is responsible for the enhanced digit span of Chinese readers, one might expect a similar span advantage in other cultures of rote learning, such as Japanese. However, Stigler et al. (1986) reported spans for Japanese children that were comparable to their American counterparts and about two digits lower than those of Chinese children, although it is noteworthy that four of the digits 1 through 9 in Japanese have two or more syllables and four are phonologically similar (transcribed as *ichi, ni, shi,* and *hachi*), both being factors that would reduce span. The issue clearly requires further investigation.

In conclusion, our results are consistent with the claim that digit and word spans are associated with speed of articulation, but they also suggest that this effect is weaker in native Mandarin speakers, and that faster articulation does not explain their superior verbal span. It seems likely that further exploration of the basis of this cross-language effect may have interesting implications for understanding the processes underpinning short-term verbal memory.

## Footnotes

1 While our study uses Mandarin, similarly enhanced memory spans are found in other Chinese languages for which digit names may differ but appear to have broadly similar characteristics (Elliott, 1992; Hoosain & Salili, 1987). We use the term *Chinese* to refer to the general effect across studies and *Mandarin* to refer to our own study.
